# The influence of tantalum on human cell lineages important for healing in soft-tissue reattachment surgery: an in-vitro analysis

**DOI:** 10.1186/s40634-019-0210-8

**Published:** 2019-10-28

**Authors:** Edward C. A. Gee, Renato Eleotério, Laura M. Bowker, Adnan Saithna, John A. Hunt

**Affiliations:** 1 Salford Royal NHS Foundation Trust, Manchester, UK; 2 School of Science and Technology, Nottingham Trent University, Nottingham, NG11 8NS UK; 3 Sano Orthopedic Clinic, Kansas City, Kansas USA; 4 Kansas City University of Medicine and Biosciences, Kansas City, MO USA

**Keywords:** Tantalum, Tendon, Soft tissue, Reattachment, Fibroblasts, Osteoblasts, Stem-cells

## Abstract

**Background:**

Porous tantalum is currently used in orthopaedic surgery for a variety of indications including soft tissue re-attachment. However, the clinical results have been variable and a previous laboratory study has suggested that tantalum may actually inhibit chick tendon fibroblasts. The influence of tantalum on human cell-types involved in soft tissue re-attachment has not been defined.

**Methods:**

Human fibroblasts, human osteoblasts and human mesenchymal stem cells were plated on glass cover slips, half of which were coated with tantalum. Cell numbers were assessed at 1, 2, 7 and 14 days using Cyquant® assay. Cell adhesion and morphology were assessed using light microscopy at 7, 14 and 28 days. To reduce the effect of an expected rate of error, *n* = 4 was utilised for each cell type and the experiment was repeated twice.

**Results:**

Statistically similar numbers of human osteoblasts and human mesenchymal stem cells were present at 14 days on tantalum-coated and uncoated glass cover slips, revealing no inhibitory effect on cell proliferation. More than double the number of human fibroblasts was seen on tantalum-coated cover slips at that time point (compared to controls), which was statistically significant (*p* < 0.0001). Morphological assessment revealed normal cell spreading and adhesion on both substrates at all time points.

**Conclusions:**

In vitro study demonstrates that Tantalum causes a significant increase in the proliferation of human fibroblasts with no quantifiable negative effects seen on fibroblast behaviour after 28 days culture. Furthermore, tantalum does not exert any inhibitory effects on the proliferation or behaviour of human osteoblasts or human mesenchymal stem cells. Tantalum could be an appropriate biomaterial for use in situations where soft tissue requires direct reattachment to implants and may stimulate soft tissue healing.

## Introduction

Porous tantalum has favourable chemical and mechanical properties for use as an orthopaedic biomaterial. Its trabeculated structure is fabricated by depositing vaporised tantalum onto a polymerised carbon foam skeleton. (Zardiackas et al., 2001) The porous structure has a Young’s modulus of elasticity that is close to cortical bone. (Levine et al., 2006) Tantalum exhibits excellent osseointegration and as a result has an established track record in arthroplasty surgery, where its main indication is to address bone loss, with vascularised bone growing through the interconnected pores in as little as 8 weeks. (Black, 1994) The potential for porous tantalum to act as a site for the direct reattachment of soft tissues such as tendons and ligaments is also of interest, but less well studied.

With an ageing global population and a higher expectation of function, the demand on revision and salvage arthroplasty implants is increasing. Loss of bone stock can include native soft tissue attachment sites (e.g. abductor mechanism to greater trochanter), affecting implant stability, joint function and survivorship of the implant. In these circumstances, (when the native attachment is no longer present) soft-tissues may require direct reattachment to a weight bearing prosthesis and porous tantalum is currently being used in this role. However, the clinical outcomes of soft-tissue re-attachment to tantalum are less well studied and perhaps more variable than its ability to osseointegrate.

Canine supraspinatus reattachment studies have shown promising results with restoration of function, stability and locomotion, (Reach et al., 2007; Itälä et al., 2007; Bobyn et al., 1982; Higuera et al., 2005; Inoue et al., 1995) but despite this, Reach et al. found that the quality of the interface between soft-tissues and tantalum implants was ‘histologically poor’ and associated with low mechanical strength. (Reach et al., 2007) Clinical human studies are limited in number but have reported restoration of collateral ligament stability around the knee, and abductor function of the hip, after direct reattachment to tantalum implants in small numbers of patients. (Chalkin & Minter, 2005; Kwong & Lin, 2010; Holt et al., 2009) In contrast, tantalum patellar augments used in the management of the symptomatic post-patellectomy knee, have met with almost universal failure, unless some residual bone stock was present. (Jordan et al., 2014; Ries et al., 2006; Tigani et al., 2009; Kwong & Desai, 2008)

In order to better understand the relationship between soft-tissue and this material, laboratory studies have investigated the cellular effects of tantalum, demonstrating increases in murine osteoblast proliferation, (Ninomiya et al., 2015; Sagomonyants et al., 2011) canine chondrocyte activity (Gordon et al., 2005) and the phagocytic capacity of leukocytes. (Schildhauer et al., 2009) It is the behaviours of fibroblasts and mesenchymal stem cells in contact with tantalum, however, that are presumed to be more important to the success of soft tissue reattachment. Animal fibroblast studies found that phagocytosis of particulate tantalum debris inhibited fibroblast proliferation and, in high enough concentrations, was cytotoxic, (Plenk, 1980; Mostardi et al., 1997) but the effect was not material specific. Another study found the presence of a porous tantalum block inhibited chick tendon fibroblast activity, reducing the cells’ capacity to adhere and produce collagen. (Jordan et al., 2014)

Despite the currently available literature, evidence of the behaviour of human cells in contact with tantalum is limited. The aim of this study was to examine the effects of tantalum on the proliferation and behaviour of human cell types involved in tissue healing and reattachment of soft tissues, namely human fibroblasts (HFs), human mesenchymal stem cells (HMSCs) and human osteoblasts (HOBs).

## Materials and methods

Circular, glass cover-slips (ø = 13 mm, SLS Ltd) were used to provide a flat substrate with minimal nanotexture. All slides were cleaned (oxygen plasma at 50w for 2 min) and half were coated with vaporised, ‘commercially-pure’ tantalum at a thickness of 50 nm (125 mA Emitch K575x Turbo Splutter Coater, LOT-QuantumDesign Ltd). All cover slips were sterilised in 70% ethanol inside a laminar flow cabinet. 24-well plates were prepared with a single coverslip of either uncoated glass (UG) or tantalum-coated (TC) glass in the bottom of each well.

Three cell types were studied; HOBs (primary derived osteoblasts from distal femur), HFs (human fibroblasts) and HMSCs (C-12972). Cells were plated in each well on either uncoated glass or tantalum-coated glass coverslips with DMEM culture medium (supplemented with 10% Foetal Calf Serum and 1% penicillin/streptomycin), which was refreshed every 2 days. In every experiment *n* = 4 to account for an expected rate of error.

An initial value of 10,000 cells per well was chosen for all cell types, however in preliminary experiments, fibroblast confluence was seen after only 7 days on both substrates. For this reason a lower number of fibroblasts (1000) was utilised to ensure the substrate remained sub-confluent, avoiding cell-cell contact inhibition.

Cyquant® assay was performed at 1,2, 7 and 14 days culture to examine the rate of cell proliferation. Serial dilutions of known cell numbers were prepared to create a standard curve for comparison of fluorescent emissions to ascertain cultured cell numbers. This was performed three times for each cell type and an average taken. The data was submitted to normality testing and analysed by two-way ANOVA and Tukey’s pair test (*p* < 0.05). For ease of reading the data in tabulated form average values were rounded to the nearest hundred.

Each cell type was removed from culture and fixed (2% paraformaldehyde fixative solution) to enable morphological analysis using light microscopy at 7, 14 and 28 days culture. The entire experiment was repeated twice to ensure correlation.

## Results

### Human osteoblasts (HOBs)

**Fig. 1:**
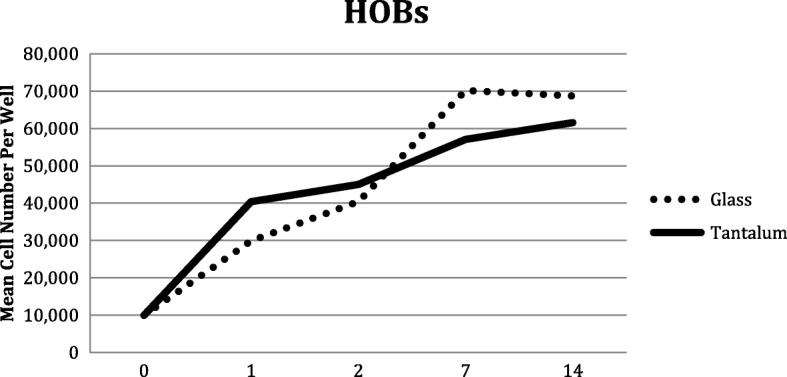
Human Osteoblast (HOBs) proliferation on glass and tantalum-coated glass substrates at 0, 1, 2, 7 and 14 days culture

After 1 day of culture there was a statistically significant increase in the number of HOBs on TC substrate compared to UG (*p* < 0.0001). At 7 days the opposite was observed with a statistically significant increase in the number of HOBs on UG (*p* < 0.0001). By 14 days there was no statistical difference in the cell numbers. (Table 1*,* Fig. 1).

**Table 1:** Mean proliferated cell numbers of Human osteoblasts (HOBs), human mesenchymal stem cells (HMSCs) and human fibroblasts (HFs) cultured on glass or tantalum-coated glass substrates at 0, 1, 2, 7 and 14 days. Mean variability reported as standard deviation

	0 days	1 day	2 days	7 days	14 days
HOB on glass	10,000	29,938 (±2710)	40,397 (±1805)	70,204 (±1775)	68,723 (±159)
HOB on tantalum	10,000	40,416 (±8126)	45,013 (±1265)	57,086 (±3927)	61,618 (±1105)
HMSC on glass	10,000	22,729 (±684)	21,547 (±2045)	38,042 (±1298)	39,624 (±2704)
HMSC on tantalum	10,000	16,961 (±4809)	24,378 (±1046)	37,068 (±2596)	37,732 (±1231)
HF on glass	1000	1418 (±175)	1096 (±238)	1105 (±208)	3535 (±820)
HF on tantalum	1000	1829 (±596)	1175 (±280)	1339 (±338)	7390 (±1618)

*Mean proliferated cell numbers of Human osteoblasts (HOBs), human mesenchymal stem cells (HMSCs) and human fibroblasts (HFs) cultured on glass or tantalum-coated glass substrates at 0, 1, 2, 7 and 14 days. Mean variability reported as standard deviation*

**Fig. 2:**
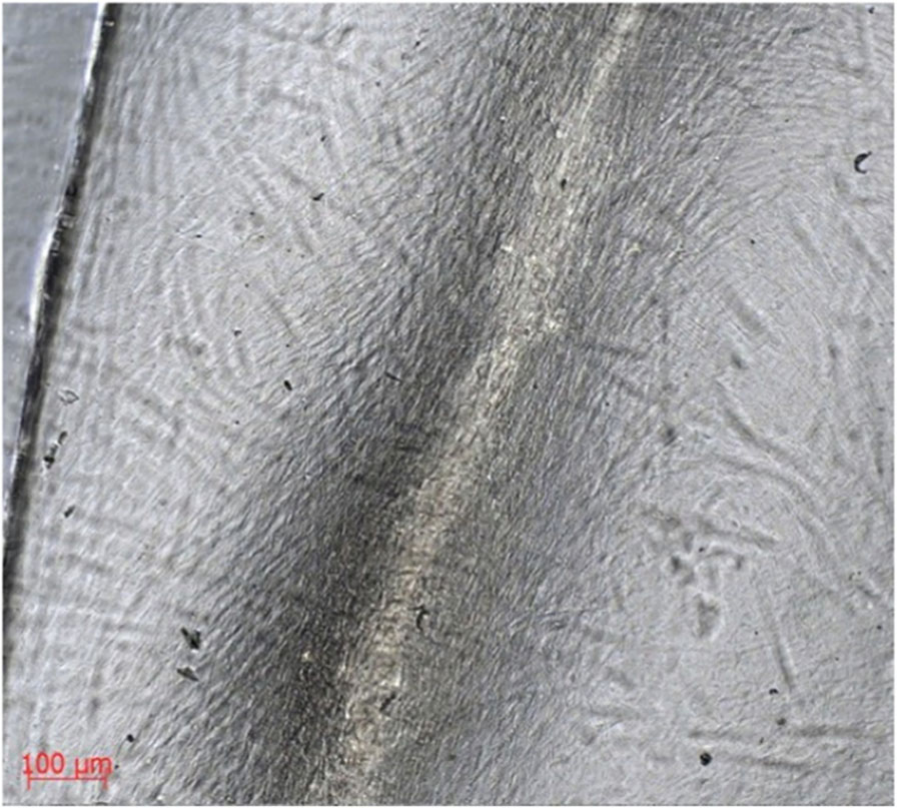
Human osteoblasts forming a bony spicule on tantalum-coated glass (1c)

Light microscopy at 7 days revealed a monolayer of HOBs on both surfaces, which was denser by 14 days. By 28 days culture, bony spicules were seen protruding from both substrates that did not wash away with passage or rinsing. (Fig. 2) There was no discernible difference between the behaviour of HOBs on the two different surfaces, the cells were adherent to the substrates with cellular projections (lamellipodia and filopodia) and peripheral spreading.

### Human Mesenchymal stem cells (HMSCs)

**Fig. 3:**
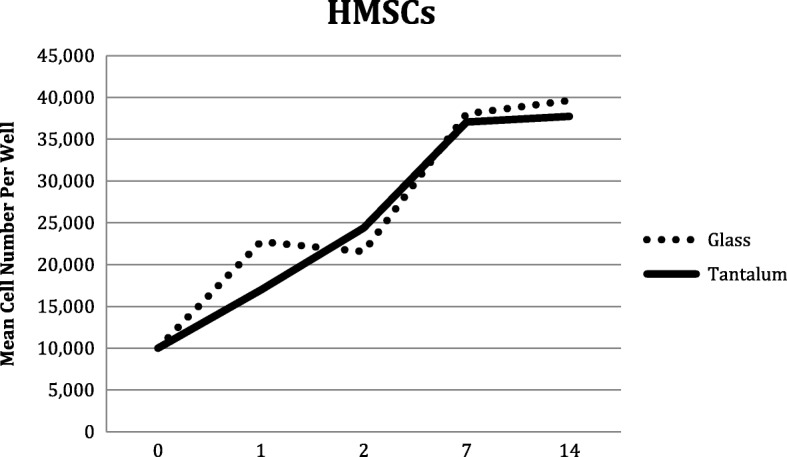
Human Mesenchymal Stem Cell (HMSCs) proliferation on glass and tantalum-coated glass substrates at 0, 1, 2, 7 and 14 days culture

Despite an initial increase in the number of HMSCs on UG substrate (*p* < 0.0001) after 1 day, the cells proliferated at a comparable rate for the rest of the time points. (Table 1*,* Fig. 3).

**Fig. 4:**
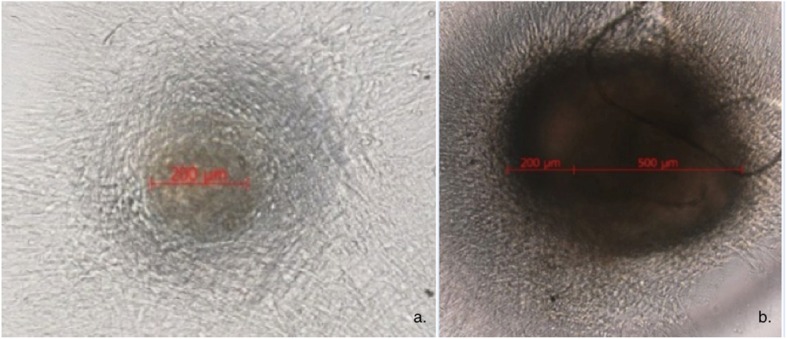
Human Mesenchymal Stem Cells at 7-day culture forming nodules on uncoated glass (**a**) and tantalum-coated glass (**b**) substrates

Light microscopy showed the cells to form a consistent, adherent monolayer on both substrates by 7 days and nodule formation was seen with a range of sizes. These nodules disappeared during cell passage and represented dense clumps of colony forming units. By 14 days the cellular nodules were seen denser and larger (500 μm compared to 200 μm) on the TC slips. (Fig. 4) By 28 days, cellular senescence was observed on both substrates.

### Human fibroblasts (HFs)

**Fig. 5:**
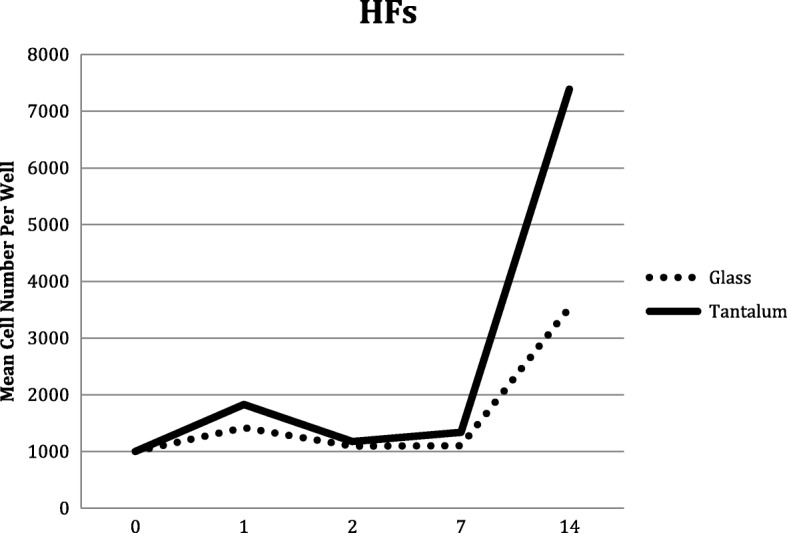
Human Fibroblast (HFs) proliferation on glass and tantalum-coated glass substrates at 0, 1, 2, 7 and 14 days culture

At this lower initial number of cells (1000) there was no statistical difference in proliferation on the 2 substrates until day 14, when there was a statistically significant increase in the number of fibroblasts on TC substrate (*p* < 0.0001). The number of fibroblasts on the TC slips was more than double (7390 ± 1618 (SD)) the number seen on the UG slips (3535 ± 820 (SD)). (Table 1*,* Fig. 5).

**Fig. 6:**
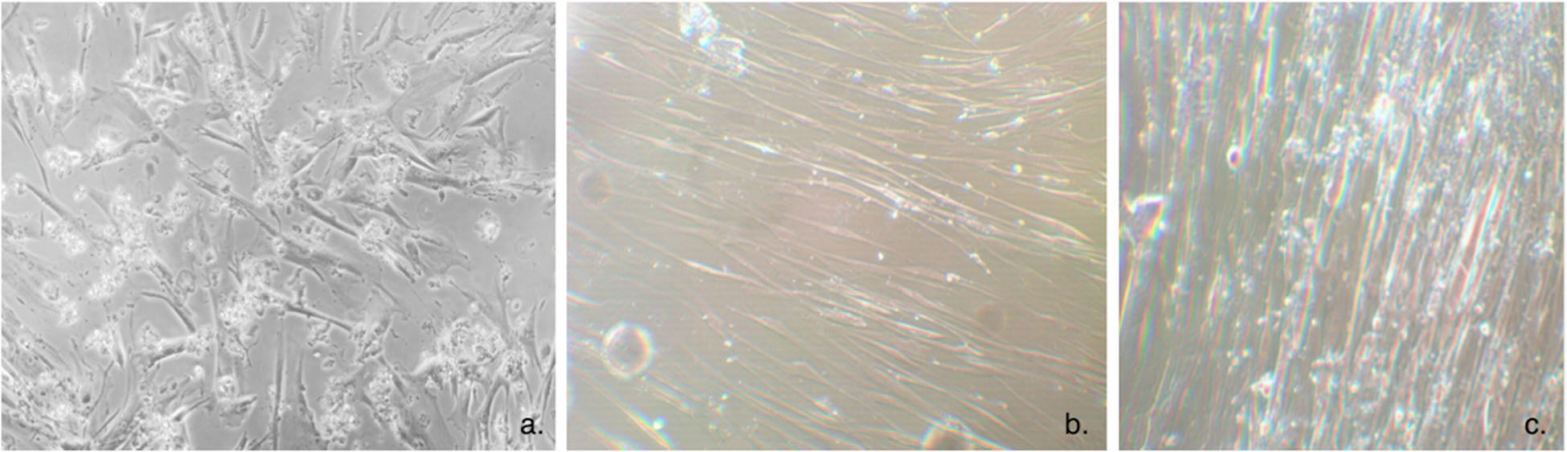
Human fibroblasts forming a dense monolayer on tantalum-coated glass, with cellular adhesion (**a**). A direct comparison of the density of the Human Fibroblast monolayer after 28 days on uncoated glass (**b**) and tantalum-coated glass (**c**)

Morphological analysis saw no recognisable difference in the behaviour of the cells on the two different surfaces. Fibroblasts were adherent to both substrates and were seen to be well-spread with thinning of the cells around the edges. (Fig. 6) A dense monolayer was formed by the cells and this became increasingly dense with time. At 28 days a direct comparison found the monolayer on the TC substrate to be visibly denser than on UG.

## Discussion

The main findings of this study were that tantalum-coated glass caused a significant increase in the proliferation of human fibroblasts after 14 days culture, with no quantifiable negative effects seen on fibroblast behaviour after 28 days culture. This finding is in contrast to previous concerns regarding the possibility of tantalum inhibiting fibroblasts. (Jordan et al., 2014) This study found no evidence of any inhibitory effect on the proliferation or cellular morphology of any of the human cell lineages studied.

Specifically, when observing the morphology of all cell lineages studied, there was no inhibitory effect seen when culturing on a tantalum-coated substrate. Tantalum’s osseointegrative success was further supported, with bony spicules seen forming on the surface of both substrates. Anchorage dependant cells, such as fibroblasts and osteoblasts, require adhesion to an underlying substrate for survival, (Baxter et al., 2002) and dense, well-attached monolayers were noted in all cultures, with the cells spreading, and forming projections (lamellipodia and filopodia).

The aforementioned study suggesting that the presence of tantalum inhibited fibroblasts used chick tendon cells and reported inhibition of cellular adhesion, with rounded, unattached cells floating in the culture medium and a reduced ability to proliferate and produce collagen. (Jordan et al., 2014) These effects may be attributable to the particular cells used, or the presence of other factors affecting cellular behaviour such as particulate debris (created by cutting blocks of porous tantalum), or the nanostructure of the material used. (Yim & Leong, 2005; Lim & Donahue, 2007; Barr et al., 2009)

When reviewing the porous material’s ability to integrate with different tissues there are more factors to consider than purely the metal element used. These include the structures porosity with large interconnecting pores providing potential for capillary ingrowth to support vascularised tissue, and also the nanostructure which has been reported to be stimulatory to certain cell types. (Plenk, 1980; Yim & Leong, 2005; Lim & Donahue, 2007; Barr et al., 2009) The macrostructure of the surface produces a coefficient of friction that can provide initial interference fixation and therefore stability for ingrowth of friable soft tissues.

Human studies, although limited, have met with some successes. Promising results were seen with functional restoration after reattachment of the hip abductors and collateral ligaments around the knee, however, these prostheses utilised compressive clamps to reattach the tendon/ligament, which may have allowed functional restoration without histologically adequate soft tissue integration into the porous structure for medium term results. (Chalkin & Minter, 2005; Kwong & Lin, 2010; Holt et al., 2009) The, almost, universal failure of patella augments in the absence of host bone may be more likely attributable to the high mechanical shear stresses at this tissue-implant interface and a poor biological environment created by multiple revision surgeries, (Jordan et al., 2014; Ries et al., 2006; Tigani et al., 2009; Kwong & Desai, 2008; Gee et al., 2016) than previous suggestions that tantalum may cause fibroblast inhibition (Jordan et al., 2014).

Despite previous concerns of tantalum causing fibroblast inhibition (from a single study using chick tendon fibroblasts) (Jordan et al., 2014), this new evidence suggests that the reverse is true. The presence of tantalum itself does not inhibit the human cells required for tissue healing (HOBs, MSCs or HFs) and actually exerts stimulatory effects on HF proliferation by 14 days. With this new information, we have an improved understanding of the likely causative factors in the success or failure of this biomaterial and can discard concerns of cellular inhibition by tantalum itself. New manufacturing processes or bio-manipulation could be utilised to enable novel methods and applications of directly reattaching soft tissues to a tantalum implant with restoration of a more natural histological attachment and a more predictable outcome.

## Conclusion

In vitro studies demonstrated that tantalum significantly increased the proliferation of human fibroblasts in direct contact with no quantifiable negative effects seen on fibroblast behaviour up to 28 days culture. Furthermore, tantalum did not exert any inhibitory effects on the proliferation or behaviour of human osteoblasts or human mesenchymal stem cells. Tantalum could be an appropriate biomaterial for use in situations where soft tissue requires direct reattachment to implants and may stimulate soft tissue healing at tissue interfaces.

## Data Availability

The datasets generated and/or analysed during the current study are not publicly available, but are available from the corresponding author on reasonable request.

## Abbreviations

DMEM: Dulbecco’s Modified Eagle Media
HF: Human fibroblast
HMSC: Human mesenchymal stem cell
HOB: Human osteoblast
PBS: Phosphate buffered saline
TC: Tantalum coated glass cover slip
UG: Uncoated glass cover slip
